# Pulley ring suture increases shoulder stability after biceps tendon transposition

**DOI:** 10.3389/fsurg.2025.1437814

**Published:** 2025-02-24

**Authors:** Duzheng Zhang, Jiezhou Wu, Liyun Zhang, Qiyun Chen, Chao Wang, Ming Cai, Ruijun Cong

**Affiliations:** ^1^ Department of Orthopaedics, Shanghai Tenth People’s Hospital, Tongji University School of Medicine, Shanghai, China; ^2^ Department of Imaging, Shanghai Changzheng Hospital Affiliated to Naval Military Medical University, Shanghai, China

**Keywords:** Pulley ring, shoulder arthroscopy, superior labrum anterior and posterior lesions, SLAP lesions, modified double-row biceps tenodesis

## Abstract

**Objective:**

Modified double-row biceps tenodesis (MDBT) has been proved to be effective in treating slap injuries, but the impact of closing the rotator cuff gap remained postoperatively (the Pulley ring repair) on the long-term shoulder function and stability has not been conclusively investigated.

**Methods:**

A retrospective study was conducted on 157 patients with isolated unilateral type II SLAP lesions treated with MDBT from January 2019 to January 2023. 77 patients were without the Pulley ring repair (group A) and the remaining 80 patients were with the Pulley ring repair (group B). *T*-test, Anova, and chi-square analyses were used to compare the two groups for any statistical differences in the 37 variables. Lasso regression was used to screen and analyze the variables, assess risk, and construct a predictive assessment model.

**Results:**

There were significant differences between the two groups at 1 year in ASES and UCLA score (*p* < 0.01). Risk factors screened by LASSO regression were five including Grouping information, Mean T value of bone mineral density, and 3 Months post operation's UCLA score. These statistically significant variables and their coefficients were used to build a generalized linear equation and selected to build the final model including Grouping information (OR:1.61E + 01;95% CI:5.8854–52.5200), Mean T value of bone mineral density (OR:6.95E + 00;95% CI:2.4695–25.6797) and 3 Months post operation UCLA score (OR:1.77E + 00;95% CI:1.4810–2.2539).

**Conclusions:**

Compared with the traditional MDBT, the modified MDBT is more effective in improving the long-term function of the glenohumeral joint and better restoring the biological state of the glenohumeral joint.

## Highlights

• This study took a rigorous analytical approach to demonstrate that modified MDBT has a significant effect on the long-term function of the shoulder.

• The modified MDBT better facilitated glenohumeral joint closure.

• It does not statistically significantly increase in operative time and does not increase the risk of perioperative complications.

## Introduction

Shoulder arthroscopy is an important tool in the management of superior labrum anterior and posterior lesions (SLAP lesions) and has been recommended by sports medicine specialists in recent years (1, 2). However, its effectiveness at the clinical treatment remains controversial (3). Superior labrum anterior and posterior lesions was proposed by Snyder in 1990 and initially classified into 4 types, later expanded to 10 (4, 5). Different types of SLAP injuries are treated with different strategies, and the therapeutic effects vary according to the patient's age and activity level. Among these, type II SLAP is the most common (6). It accounts for 55% of all SLAP injuries. It is characterized by persistent pain and partial limitation of movement due to avulsion of the long head tendon of the biceps brachii (LHBT) and the superior labrum from the glenohumeral joint and superior border, leading to chronic shoulder pain and disability in the long term, especially in people with a lot of upper extremity overhead movements, such as pitchers and volleyball players (7, 8).

Patients with simple type II SLAP injuries that do not respond to conservative management are treated surgically with a variety of surgical protocols. 15 procedures are available, including arthroscopic SLAP repair, arthroscopic SLAP repair combined with LHBT tenotomy, simple LHBT tenotomy, and LHBT tenotomy (9–12). Each of these approaches varies depending on the patient's recovery process and rehabilitation strategy. Despite the increasing number of such procedures, there is no consensus on the optimal surgical approach or management strategy for type II SLAP injuries (13, 14). Modified double-row biceps tenodesis (MDBT) is a surgical procedure proposed by our team in 2014 and has been shown in clinical trials to be effective in the treatment of type II SLAP injuries (15).

MDBT has been shown to be effective in improving long-term joint function compared with the upper glenoid labral suture technique alone in a number of follow-up studies of more than 20 months. However, long-term shoulder function is less well restored in younger, more athletic patients than in middle-aged patients with less exercise. Also, the recurrence of shoulder discomfort was common in the long-term follow-up. This discomfort was not severe pain or dysfunction, and was mostly transient during relatively strenuous exercise. We speculate that the disruption of the vacuum-negative environment of the glenohumeral joint may be responsible for this discomfort.

MDBT consists of the following steps: firstly, cutting the LHBT within the joint near the superior glenoid labrum; secondly, retracting the LHBT extra-articularly through the rotator cuff gap; thirdly, loosening the LHBT tendon membrane; and finally, securing the LHBT in the bicipital groove. During the operation, we noticed that when the LHBT is pulled out of the rotator cuff gap (the Pulley ring), a tube is left under the Pulley ring. There is not any study conforming whether the tube would close itself, which means it is uncertain whether the vacuum-negative environment in the rotator cuff gap could be restored in the long term after surgery. Therefore, it is unclear whether closing the rotator cuff gap (also known as the Pulley ring repair) contributes to creating a vacuum structure in the glenohumeral cavity, which subsequently increases joint stability in the distant postoperative period after MDBT. The aim of this study was to investigate the effect of closing the rotator cuff gap or not on shoulder function and shoulder stability in the long term after MDBT by reviewing 157 patients with MDBT treated at our center from January 2019 to January 2023, with 1-year postoperative follow-up.

## Methods

### Inclusion and exclusion criteria

The inclusion criteria were carried out as follows: (i) clinical symptoms, physical examination and magnetic resonance imaging suggested the presence of type II SLAP lesions; (ii) acceptance of MDBT operation; and (iii) the diagnosis should ultimately be verified at arthroscopy.

The exclusion criteria were carried out as follows: (i) associated with partial or full-thickness rotator cuff tears; (ii) previous surgery for the same shoulder; (iii) professional overhead athletes; (iv) severe arthritis of the glenohumeral or acromioclavicular joints; (v) intra-articular chondral damage; or (vi) associated with other shoulder joint chondral diseases.

The study was approved by the ethics committee of Shanghai 10th hospital and has been conducted in accordance with the principles set forth in the Helsinki Declaration.

### Patient enrolment

There were 157 patients with isolated type II SLAP lesions treated by MDBT involved in this study, from January 2019 to January 2023. All patients were unilateral SLAP injury. A total of 77 patients (male/female = 37:40) were without the Pulley ring repair (Group A), with a mean follow-up of 12.8 ± 2.3 months (range, 12–14 months). The remaining 80 patients (male/female = 39:41) were with the Pulley ring repair (Group B), and the mean followed-up was 12.6 ± 3.8 months (range, 12–14 months). All the operations were completed by the same group of surgeons.

### Outcome measures

For all patients enrolled in this study, the preoperative data and 3-day postoperative data were recorded and assessed through physical examination and medical records, at the 3month after operation and final follow-up, other postoperative data were recorded through outpatient follow-up. Preoperative assessments with the Age, Gender, Height, Weight, BMI, Tendon calcification, Fasting Blood Sugar, Glycosylated Hemoglobin Value, Hypertension, Cervical Spondylosis, Aspirin inhibition rate, Clopidogrel inhibition rate, Inactivated Platelet function, Uninhibited Platelet function, Uninhibited Platelet activated function, Total Cholesterol, Triglyceride, High Density Lipoprotein, Low Density Lipoprotein, 25 Hydroxy Vitamin D, Mean T value of bone mineral density, R value of Thrombus Elastogram, κ value of Thrombus Elastogram, solidificated angle of Thrombus Elastogram, Clot strength of Thrombus Elastogram, operative time, University of California, Los Angeles (UCLA) score, and American Shoulder and Elbow Surgeons (ASES) and postoperative assessments with the UCLA score and ASES score were compared between the two groups. Additional outcome measures included patient satisfaction, the time to return to previous activities, workers’ compensation status, and postoperative complications. The baseline characteristics for the patients are summarized in Table 1.

**Table 1:** Patient characteristics in both groups.

	Group A^a^	Group B^b^	Statistic	*p* value
Age(years)	63.1 ± 19.2	58.3 ± 21.6	1.48	0.142
Gender(male/female)	37/40	39/41	0	1
Height(cm)	161.9 ± 9.3	163.2 ± 8.2	−0.93	0.354
Weight(kg)	68.3 ± 14.4	65.8 ± 12.9	1.13	0.261
BMI(kg/m^2^)	26 ± 4.9	24.7 ± 4.6	1.72	0.088
Surgery on the dominant arm	42	39	0.32	0.571
Tendon calcification	2	3	0	1
Hypertension	26	27	0	1
Cervical Spondylosis	3	5	0.09	0.758
History of shoulder joint trauma	1	2	0	1
Fasting blood sugar (mmol/L)	5.9 ± 2.1	5.6 ± 1.9	0.84	0.404
Glycosylated hemoglobin value (mmol/L)	5.7 ± 1.7	5.4 ± 1.7	1.04	0.301
Aspirin inhibition rate (%)	0.7 ± 0.2	0.8 ± 0.2	−0.76	0.447
Clopidogrel inhibition rate (%)	0.6 ± 0.2	0.7 ± 0.2	−0.9	0.371
Inactivated Platelet function (mm)	5.7 ± 1.5	5.7 ± 1.4	0	0.997
Uninhibited Platelet function (mm)	42.1 ± 6.1	42.1 ± 5.5	0.02	0.986
Uninhibited Platelet activated function (mm)	20 ± 3	20 ± 2.7	0.03	0.975
Total Cholesterol (mmol/L)	5.5 ± 1.4	5.5 ± 1.4	−0.04	0.965
Triglyceride (mmol/L)	2.1 ± 0.7	1.9 ± 0.7	1.36	0.177
High Density Lipoprotein	1.3 ± 0.2	1.3 ± 0.2	2.38	0.019
Low Density Lipoprotein	3.4 ± 0.5	3.4 ± 0.5	0.42	0.678
25 Hydroxy Vitamin D (ng/ml)	22.2 ± 9.3	23.4 ± 8.4	−0.88	0.378
Mean T value of bone mineral density	−0.3 ± 0.5	−0.3 ± 0.4	−0.55	0.586
R value of thrombus elastogram (min)	7.8 ± 1.8	7.8 ± 1.6	0.03	0.978
*κ* value of thrombus elastogram (min)	1.8 ± 0.6	1.8 ± 0.6	0.49	0.627
Solidificated angle of thrombus elastogram (deg)	63 ± 8	61.1 ± 7.3	1.55	0.124
Clot strength of thrombus elastogram (mm)	58.8 ± 4.9	57.3 ± 4.8	1.82	0.071
ASES pre operation	26.5 ± 11.6	24.3 ± 12.2	1.16	0.246
UCLA score pre operation	10.1 ± 3.2	9.8 ± 3.2	0.67	0.506

Abbreviations: ASES, American shoulder & elbow surgeons score; SD, standard deviation; UCLA, University of California Los Angeles score.

^a^ MDBT without the Pulley ring repair group.

^b^ MDBT with the Pulley ring repair group.

### University of California, Los Angeles (UCLA) scores

Function and pain were evaluated independently, in UCLA Scores, with a scale of 0–10. The score of 1 represented the worst possible score, while 10 represented the best. The range of motion of the shoulder, muscle strength, and patient satisfaction were also included in the scoring system and each given a maximum of 5 points. So, this modified UCLA shoulder scoring system had a total of 35 possible points. Results were classified as excellent (34–35), good (28–33), fair (21–27), and poor (20 and below).

### American shoulder and elbow surgeons (ASES) scores

This was a converted percentage system in which the patient evaluated the portion of pain (50%) and accumulated daily activities (50%) as the scoring component. Patients self-assessed for pain, stability and daily activities, while the doctor evaluated the sections for activity, physical signs, strength tests, and stability. Although historically, this was based on the patient and physician's subjective and objective comprehensive evaluations, the current scoring is solely based on the patient's subjective score including pain (50%) and living function (50%), with a maximum score of 100. The higher the score, the better the shoulder result.

### Surgical technique

**Figure 1:**
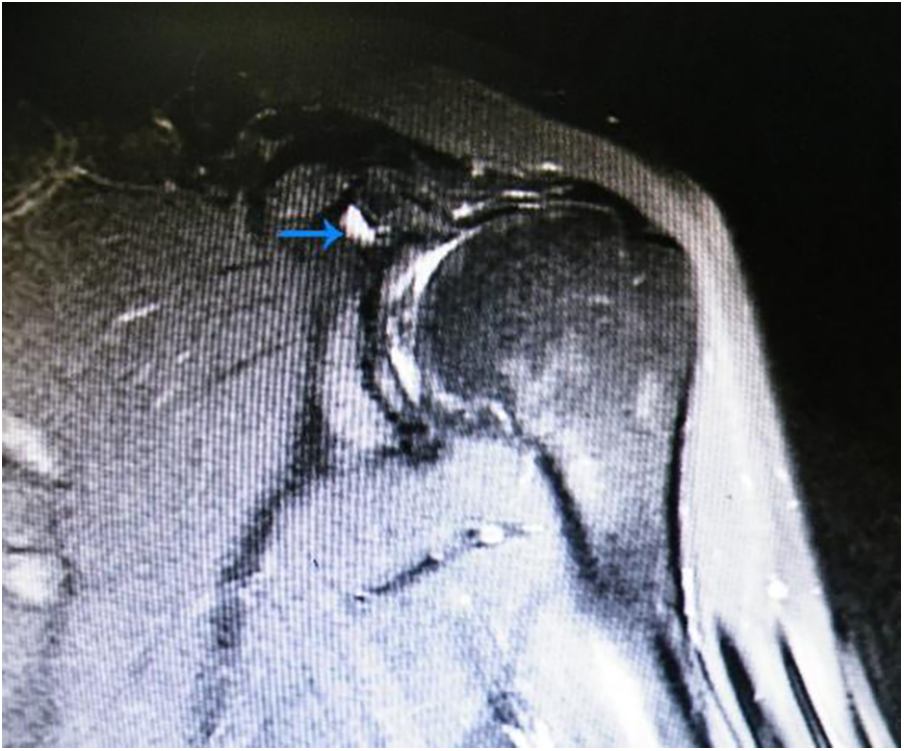
Preoperative radiographic images. The blue arrows indicate the type II superior labrum anterior and posterior (SLAP) lesion.

**Figure 2:**
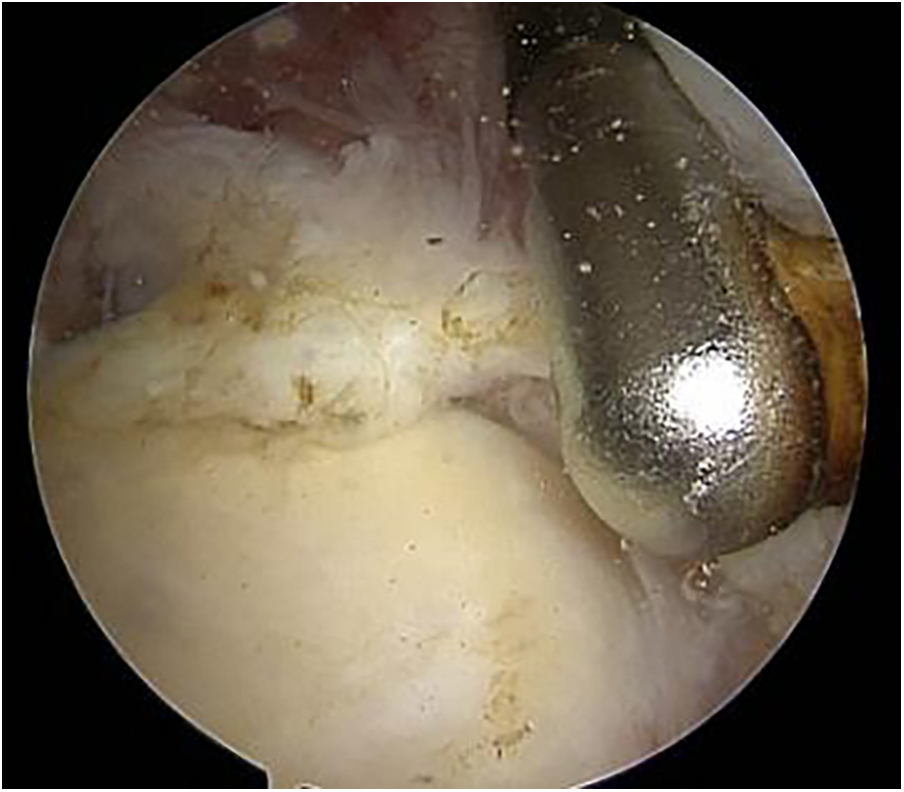
Intraoperative arthroscopic photograph showing type II superior labrum anterior and posterior (SLAP) lesion.

During biceps tenodesis, the diagnostic arthroscopic evaluation was initially carried out through a standard posterior portal (Figures 1, 2). The evaluation of the diagnosis of type II SLAP lesion was defined by the probe from the anterior viewing portal. After examining the superior labrum, the spinal needle was used to fix the LHBT inside the articular cavity. To maintain the position of the LHBT and to facilitate the accurate positioning of the LHBT in the bicipital groove, the arrival angle of the spinal needle should be adjusted with the method described by our previous study. The LHBT was severed on the superior labral attachment, and the integrity as well as stability of the superior labrum were examined immediately. To create a stable and smooth surface, the inflamed soft-tissue and synovium on the bicipital groove were cleaned out and the LHBT were released. After determining the tension and direction of the LHBT, two suture anchors were used to fix it. The superior anchor was used for a suture loop knot, and the inferior anchor was used for the loop knot, as described in our previous study.

**Figure 3:**
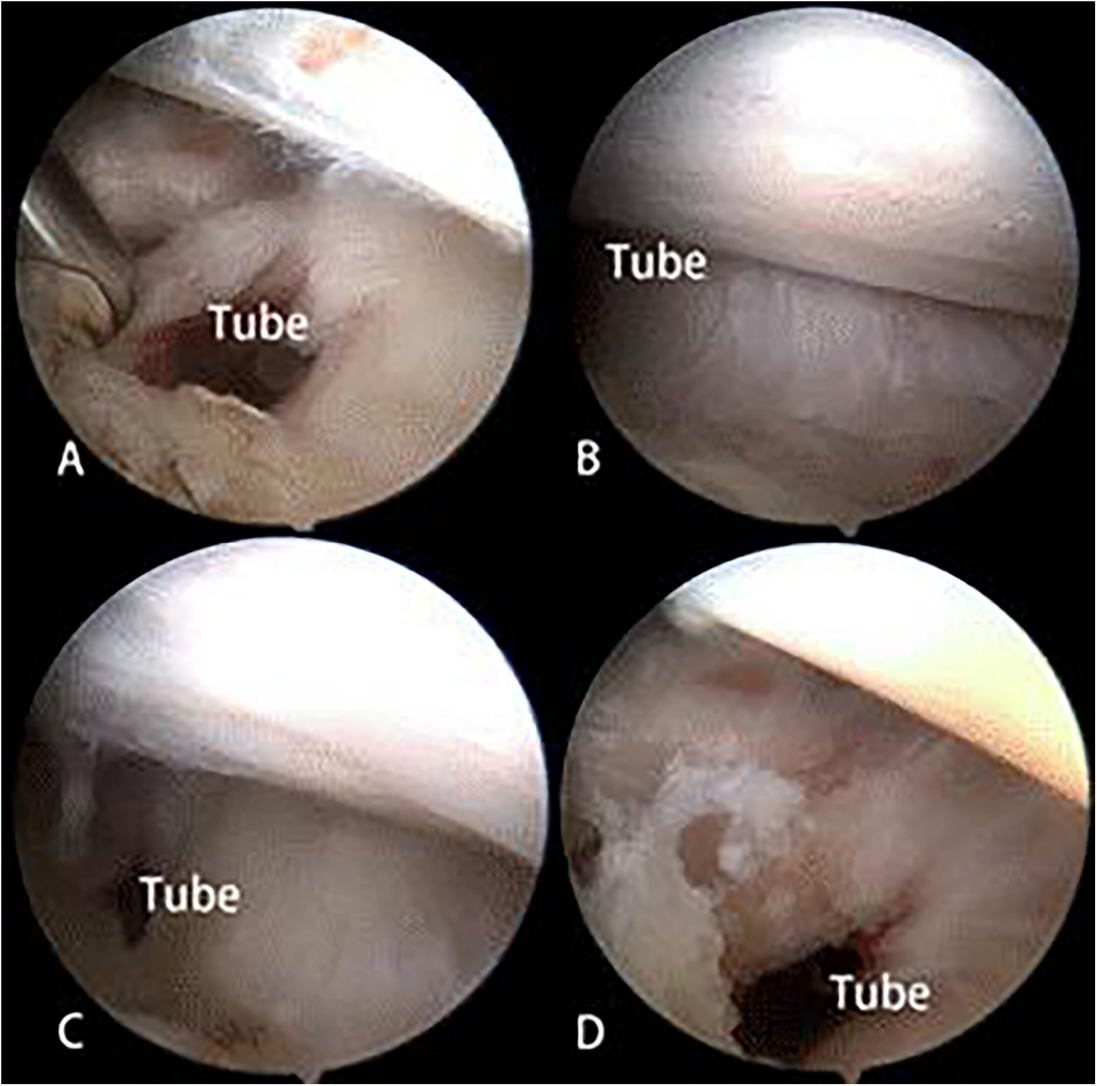
The tube outside of the joint was left between the pulley ring and the bottom of the groove, connecting the inside and outside of the articulatio humeri. **(A**–**D)** The tube in some cases.

**Figure 4:**
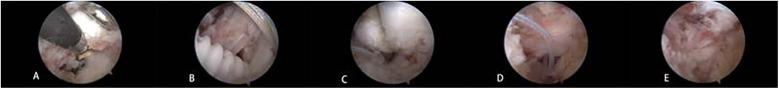
The process of the tube closure: **(A)** A suture anchor was fixed into the bone structure at the bottom of the groove. **(B)** A lasso guided a limb of the tail line of the suture anchor across the supraspinatus tendons at a point 0.5 cm from the edge of the tendons. **(C)** The lasso guided another limb of the tail line across the subscapular tendons at the similar point as last step. **(D,E)** The tube was closed after the knot was tied.

**Figure 5:**
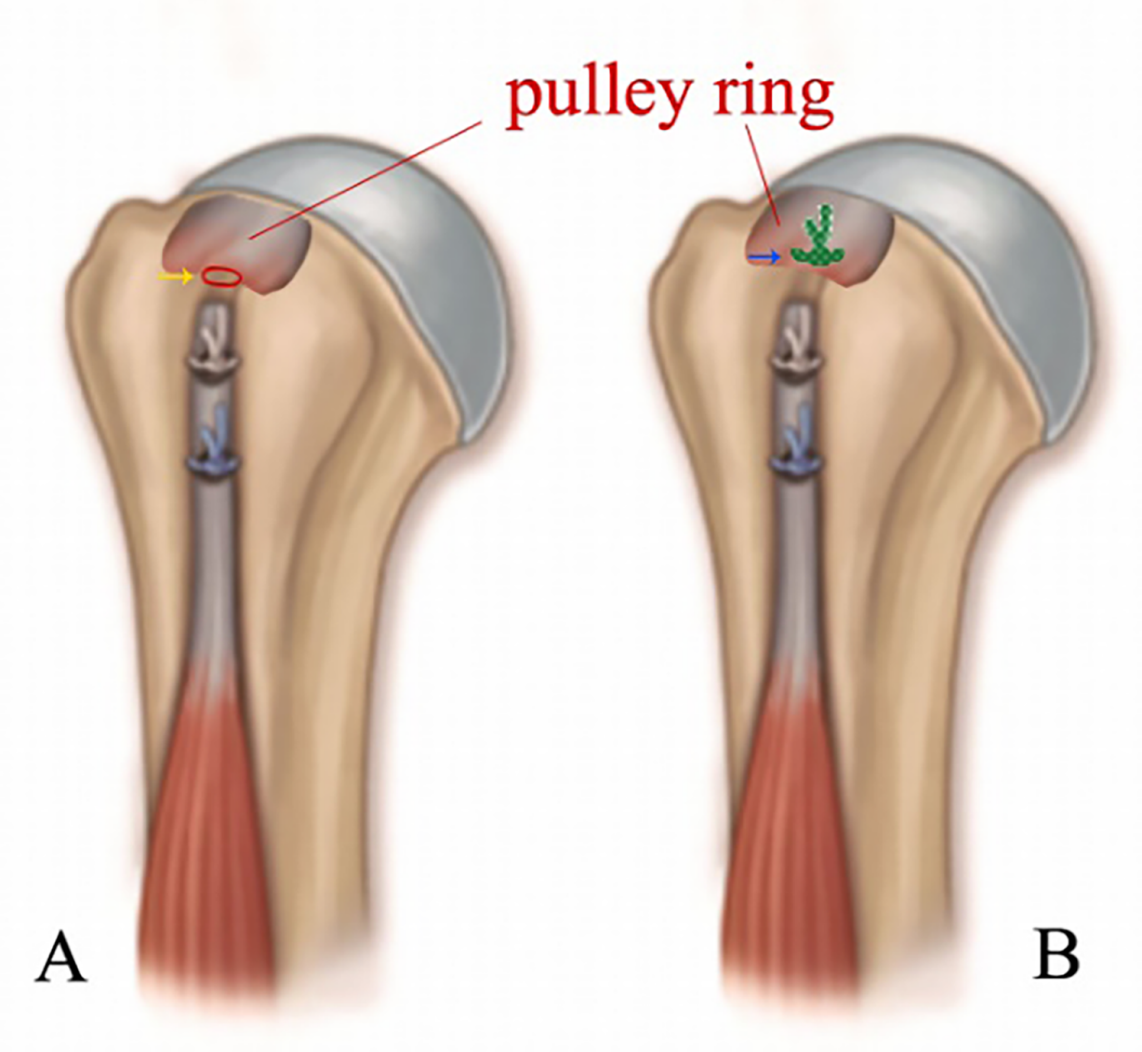
Schematic diagram of the arthroscopic. MDBT with and without the Pulley ring repair: **(A)** Biceps Tendon was trans posited to intertubercular sulcus and a tube was left between the Pulley ring and the bone surface (yellow arrow); **(B)** An anchor was implanted under the Pulley ring, and the tail lines across the Pulley ring to close the tube.

Then the Pulley ring of the patients in group B was repaired with another suture anchor. The groove, also known as the intertubercular sulcus, in which the LHBT extends from the outside of the joint to the inside at the superior and anterolateral caput humeri, between the supraspinatus tendons and the subscapular tendons. The tendons migrate and transform into a membranous structure, also known as the Pulley ring, which covers the groove surface. After the LHBT was transposed and sutured in the groove, which is part of the intertubercular sulcus, a tube outside of the joint was left between the Pulley ring and the bottom of the groove, connecting the inside and outside of the articulatio humeri (Figure 3). The tube was closed using a suture anchor that was fixed into the bone structure at the bottom of the groove. A lasso guided one limb of the tail line of the suture anchor across the supraspinatus tendons at a point 0.5 cm from the edge of the tendons. Also, the lasso guided another limb of the tail line across the subscapular tendons at the similar point as last step. The tube was closed after the knot was tied (Figure 4). Group A did not close the tube (Figure 5).

## Ultrasonographic measurement

**Figure 6:**
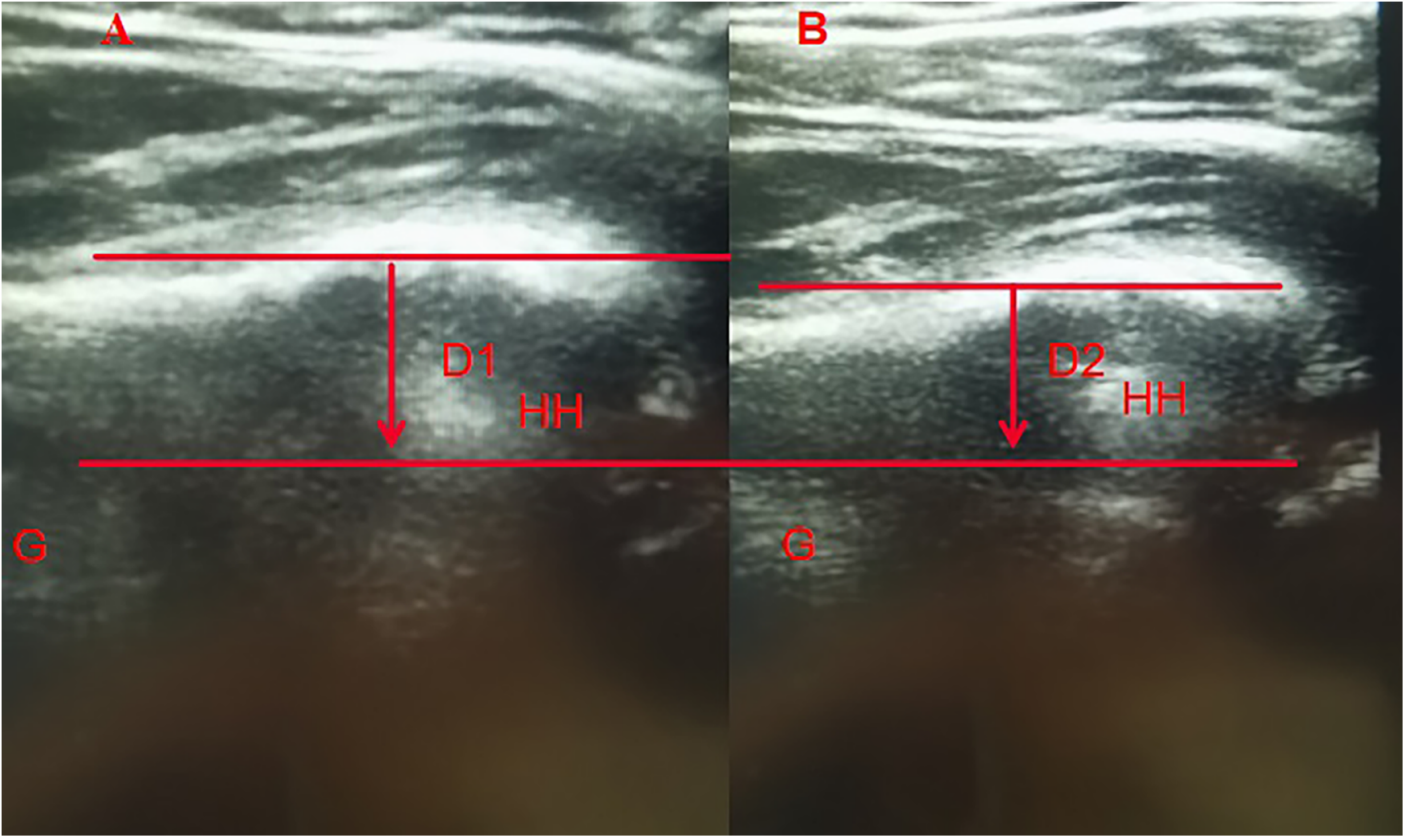
Ultrasonographic images figure **(A)** does not give a distraction and figure **(B)** gives a 40-N distraction. Two parallel lines were drawn through the posterior edges of glenoid and HH. The shortest distance between the 2 lines was measured both without (*D1*) and with (*D2*) 40-N distraction. Anterior HH translation was calculated by subtracting *D2* from *D1*. HH, humeral head; G, glenoid.

Ultrasonographic evaluation was performed immediately after surgery. The forearm was fixed with an arm positioner in the beach-chair position, and the ultrasonographic transducer was located at the posterior part of the shoulder to visualize the humeral head and glenoid rim at the level of interval between the infraspinatus tendon and teres minor tendon. The upper arm was drawn anteriorly with a 40-N force at 0°, 45°, and 90° of shoulder abduction with neutral rotation. The distance from the posterior edge of the glenoid to that of the humeral head (△ d) was measured using ultrasonography with and without anterior force. Anterior translation was defined by subtracting the distance with anterior force from the distance without anterior force. To measure distance, 2 lines were drawn parallel to the posterior edge of the glenoid and humeral head. The shortest distance between the 2 lines was measured without (D1) and with 40-N distraction (D2). Negative values were assigned when the posterior edge of the humeral head was anterior to that of the glenoid. △ d was defined by subtracting D2 from D1 (Figure 6) (16).

### Postoperative rehabilitation

Patients did not require a fixation device and should carry out the perioperative rehabilitation training under the guidance of rehabilitators, as described in our previous study (15). No complications were reported in either group.

### Statistical analyses

Continuous variables were expressed as means and standard deviations. All statistical analyses were conducted using the language R for Windows, (R version 4.3.1). Two-tailed t-tests or Mann–Whitney *U*-tests were used to compare the differences between groups, and Anova was used to analyze the differences in scores between different follow-up time points, such as in the ASES and UCLA scores. The chi-square test was used to calculate *p* values for classified data and expressed in frequencies and percentages, *p* values of less than 0.01 were considered significant. The correlation between all the variants was calculated using Spearman regression analysis. The packages base, datasets, graphics, grDevices, methods, stats, utils, glmnet, ggplot2, patchwork, ggcor, and GGally were used in the Statistical Analyses.

The Least Absolute Shrinkage and Selection Operator (LASSO) method was used to reduce multivariate data and select risk factors for the final joint functional and pain score's result of MDBT. The training set used non-zero LASSO regression coefficients and the result of the last follow-up was used as the target factor. Multiple logistic regression analysis with lambda.min model was then performed on selected features from the LASSO regression model to create a predictive model. The characteristics odds ratio (OR) with 95% confidence interval (CI) and *p*-value were taken into account. The statistical significance levels are two-tailed. Sociodemographic variables were included in the model with a *p*-value less than 0.01, while disease- and treatment-related variables were included.

## Results

### Follow-up and baseline

One year follow-up was carried out for all 157 MDBT patients with isolated type II SLAP lesions (Table 1). Using ASES and UCLA scores as the primary way to consider surgical outcomes. The demographic data mentioned in the methods did not differ significantly between groups A and B. At the same time, no significant complications were found during the follow-up period.

### ASES and UCLA scores

There were significant differences between the two groups at the 1year follow-up on ASES and UCLA scores (Table 2). As in our previous study, the ASES and UCLA scores were significantly different at four time points, which were pre-operation, 3 days after operation, 3 months after operation and 1 year after operation, according to the Anova (Table 3). Further subgroup analysis, using the TukeyHSD method, confirmed that the scores were significantly different between each time point (Table 4).

**Table 2:** Comparison between two groups.

	Group A*	Group B^†^	Statistic	*p* value
3 days post op ASES score	75.9 ± 8.7	75.3 ± 8.7	0.43	0.668
3 months post op ASES score	83.9 ± 5.4	82.8 ± 6.5	1.10	0.274
1 year post op ASES score	89.9 ± 4.8	97.4 ± 3.6	−11.15	<0.01
Interval return to work	14.2 ± 4.9	14.0 ± 5.5	0.19	0.852
3 days post op UCLA score	22.3 ± 2.1	22.2 ± 2.0	0.42	0.675
3 months post op UCLA score	29.1 ± 3.0	29.2 ± 3.1	−0.20	0.842
1 year post op UCLA score	30.8 ± 3.0	33.0 ± 2.5	−5.02	<0.01
time of op	68.1 ± 12.3	72.7 ± 11.8	−2.37	0.019

**Table 3:** Anova at 4 time points.

	Df	Sum Sq	Mean Sq	F value	Pr(>F)
Factor	3	433,991	144,664	2,030	<0.01
Residuals ASES	624	44,468	71		
Factor	3	45,301	15,100	1,866	<0.01
Residuals UCLA	624	5,050	8		

**Table 4:** Subgroup analysis for the anova at 4 time points.

	UCLA scores				ASES			
	Diff	Lwr	Upr	p.adj	Diff	Lwr	Upr	p.adj
3Dop-1Yop	−9.68	−10.50	−8.85	<0.01	−18.13	−20.59	−15.68	<0.01
3Mop-1Yop	−2.75	−3.58	−1.92	<0.01	−10.36	−12.82	−7.91	<0.01
Preop-1Yop	−21.99	−22.81	−21.16	<0.01	−68.36	−70.82	−65.91	<0.01
3Mop-3Dop	6.92	6.10	7.75	<0.01	7.77	5.32	10.23	<0.01
Preop-3Dop	−12.31	−13.14	−11.49	<0.01	−50.23	−52.68	−47.77	<0.01
Preop-3Mop	−19.24	−20.06	−18.41	<0.01	−58.00	−60.45	−55.55	<0.01

## Ultrasonographic measurement

Measurement of △ d was carried out for 56 individuals in group A and 80 individuals in group B. The mean value of pulling was obtained for both groups. Then, a *t*-test was performed and it was found that there was a significant difference between the two groups’ △ d.

### Correlation analysis

**Figure 7:**
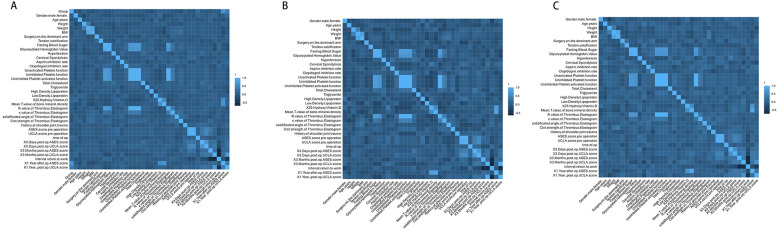
The correlations of all the variables. The diagram at the bottom left visualizes the correlation of variables. The color depth represents the degree of the correlations, the right colorimetric card of every figure represents the R-value, and the size of the square indicates the degree of confidence of the correlation, the values in the upper right are intergroup correlation coefficients. Signif. codes: *** for *p* < 0, ** for 0.01 < *p* < 0.05, * for *p* > 0.05. **(A)** The correlations of all the variables in both groups, the grouping information was treated as nominal variable. **(B)** The correlations of all the variables in group A, which is MDBT without the Pulley ring repair group. **(C)** The correlations of all the variables in group B, which is MDBT with the Pulley ring repair group.

The correlations of the 37 variables we collected for statistical analyses are shown in Figure 7. The color depth represents the degree of correlation. Part A expresses the overall correlation between the variables. ASEC and UCLA scores were the primary metrics analyzed. As shown, these two scores correlate strongly with grouping, indicating that grouping is an important correlate of the two scores. Part B and C compare the correlations between the variables within the two groups. The correlations between the variables without grouping information show different distributions between the two groups.

### Lasso regression analysis

**Figure 8:**
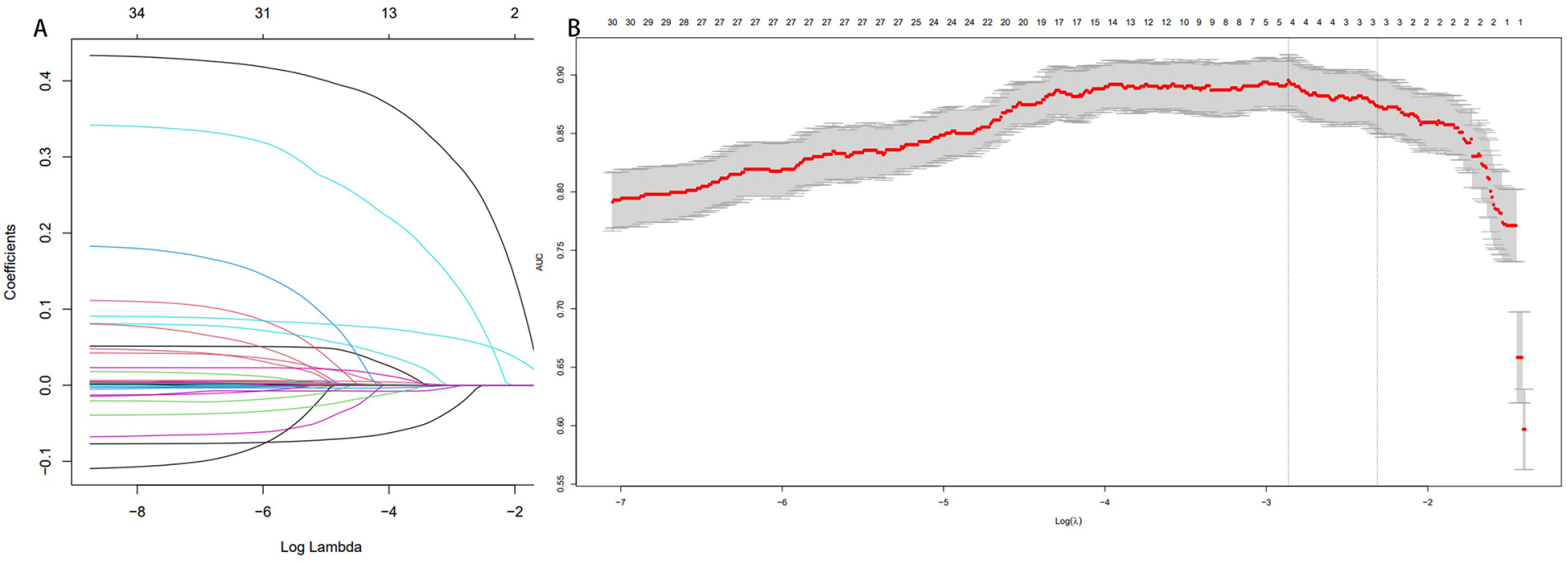
Lasso regression images. **(A)** Lasso coefficient path diagram. **(B)** Lasso regression analysis cross validation curves.

The results of the last follow up were defined as the target to carry out the lasso regression analysis. Five potential predictors were selected from 37 variables based on data from 157 patients in the training cohort and their nonzero coefficients in the LASSO regression model were also built in Figure 8. A generalized linear equation was formed using these variables and their coefficients. Three variables, including Grouping information, Mean T value of bone mineral density, and 3 Months post operations UCLA score, were confirmed to be statistically significant and were selected to build the final model. The OR value and 95% confidence interval were also presented (Table 5).

**Table 5:** The final model.

	*β* value	Screening of the predicted variables					95% confidence interval		OR value
		Estimate	Std. error	z value	Pr(>|z|)		2.50%	97.50%	
(Intercept)	−1.615744341	−18.58819	3.50196	−5.308	1.11 × 10^−7^	***	0.0000	0.0000	1.48 × 10^−9^
Grouping information	0.286231981	2.7835	0.55294	5.034	4.80 × 10^−7^	***	5.8854	52.5200	1.61 × 10^+1^
Triglyceride	−0.023847849	−0.56881	0.33042	−1.721	0.085164	.			
Mean.T.value.of.bone.mineral.density	0.124302717	2.0061	0.59002	3.4	0.000674	***	2.4695	25.6797	6.95 × 10
UCLA.score.pre.operation	−0.000503052	−0.11736	0.07358	−1.595	0.110704	.			
3.Months.post.op.UCLA.score	0.060332626	0.584	0.10617	5.501	3.79 × 10^−8^	***	1.4810	2.2539	1.77 × 10

Signif. codes: 0 ‘***’ 0.001 ‘**’ 0.01 ‘*’ 0.05 ‘.’ 0.1 ‘ ‘ 1.

## Discussion

### Theoretical advantages and technical points of the modified MDBT favoring the long-term function of the glenohumeral joint

A study on the biomechanics of the glenohumeral joint has revealed that the vacuum environment within the glenohumeral joint is essential for maintaining its stability (17). The vacuum-negative environment in the glenohumeral joint enhances the adsorption of the glenoid on the humeral head which increases the stability of the glenohumeral joint (18–20). The current concept for shoulder stability states that the glenohumeral joint is supported by a hierarchy of passive and active mechanisms: concavity, limited joint volume, adhesion and cohesion; ligamentous, capsular and bony restraints; and active muscular control (21, 22). Unsewn pulley rings can cause damage to the integrity of the vacuum environment and pressure build-up. Whereas negative pressure can be responded to by postoperative general anaesthesia conditions of △ d (16). Because of the high rate of pull-offs in Group A, △ d measurements were taken in only some patients. There was a significant difference in △ d between groups A and B, suggesting that suturing of the pulley ring was more benefitable to the recovery of negative pressure. Also, the inward stress generated by the negative pressure is an important factor in maintaining the balance of the rotator cuff force couple. Furthermore, in comparison to MDBT alone, the modified MDBT maintains the continuity of both the supraspinatus tendons and the subscapular tendons within the rotator cuff gap and preserves the integrity of the rotator cuff after the LHBT transposition, which reduces the risk of secondary rotator cuff tears in the long term. At one year follow-up, there were no complications of rotator cuff tears in either group in this study. Although the likelihood of secondary rotator cuff tears after SLAP injury is not clear, reduced shoulder stability after MDBT has been reported (23).

The structures surrounding the glenohumeral joint are complex and simultaneous destruction of multiple structures is common (19, 20, 23, 24).The SLAP injuries combined with the Pulley ring injuries and supraspinatus anterior margin microtears are usually more common (25–27). A prospective cohort study has confirmed that patients with rotator cuff injuries have a greater than 30% chance of combining SLAP injuries (28). Similarly another cross-sectional study has found that the odds of having SLAP injuries combined with supraspinatus tendon microtears are higher than the odds of not combining them (29). The superiority ratio for the probability of tear is 3.25 and the difference is statistically significant. Due to limitations in preoperative examination techniques and clinical technology, simultaneous repair of SLAP injuries, anterior supraspinatus microtears, and the Pulley ring lesions has never been fully performed (30). We believe that this is an important reason for increased postoperative pain, especially distant shoulder instability and pain, and residual shoulder dysfunction.

### Improvements in long-term function and associated factors

At the one-year postoperative follow-up, the group that switched to modified MDBT had better ASES and UCLA scores compared to the MDBT-only group. And lasso regression targeting shoulder function at one year postoperatively showed that grouping information was an independent prognostic predictor, and in particular grouping was strongly correlated with ASES, all of which confirmed that the modified MDBT had better shoulder function in the distant postoperative period. However, this functional advantage was not evident in the immediate postoperative period and did not reduce the time to return to work. Also, unmodified MDBT technique did not result in more postoperative complications. We hypothesize that the functional advantage in the distant future is related to the closure of the LHBT tunnel opening in the rotator cuff gap which ensures the acquisition of a hermetically sealed environment within the shoulder in the distant postoperative period. Several studies have confirmed the existence of this hermetically sealed negative pressure environment and the importance of it for shoulder stability (31, 32).

We also note that osteoporosis, as an independent risk factor, is an important correlate of the long-term prognosis of MDBT (33). Bone mineral density and 25 Hydroxy Vitamin D levels influence the long-term prognosis of the glenohumeral joint with or without the use of the modified MDBT (34), which is also worthy of further investigation.

### Rotator cuff gap closure as an option for MDBT is technologically advanced

MDBT has obvious advantages over traditional labral repair of the shoulder (35). The need for closure of residual tubes after LHBT translocation has not been confirmed by accurate large-scale clinical studies (36). Our study confirms that closure of the rotator cuff gap has a significant effect on improving long-term shoulder outcomes. It is an optional technique that does not require a significant increase in technical complexity or an additional learning curve for the established sports medicine practitioner. It will not statistically significantly increase in operative time. Furthermore, it does not increase the risk of perioperative complications.

It is well known that the bicipital tunnel is a confined space consisting of three distinct zones (37, 38). Although connected to the joint cavity, the bicipital groove only accumulates large amounts of fluid in cases of severe inflammation. Under normal circumstances, it is a potential gap that maintains a negative pressure environment in the joint cavity. After the LHBT is transposed beyond zone II of the bicipital groove, a tube is formed between the Pulley ring, which is formed by the continuation of the supraspinatus and subscapular tendons, and the bony structures at the base of the bicipital groove. As previously mentioned, the first technical point is to thoroughly clean the inflammatory synovial tissue at the base of the bicipital groove to clearly expose the fresh bone surface, which enables tendon-bone healing at a later stage. There is cartilage-like tissue migration or bone tissue. And if there is the cartilage-like tissue migration, a planer should be used to clean out enough bone interface to facilitate tendon healing. The second technical point is that the point of anchor placement at the base of the bicipital groove should be relatively inward. Placing the anchor closer to the humeral head can reduce the Pulley ring suture tension and better seal the canal. The third point is to avoid excessive puncture of the supraspinatus and subscapular tendons to ensure the original direction of tension in the rotator cuff and avoid postoperative pain due to changes in the direction of tendon tension.

A previous study showed 225 young, active patients undergoing SLAP repair reported a 37% failure rate and a 28% reoperation rate15 (39). Also, pain rates and in-surgery rates similar to MDBT. Modified MDBT has lower recurrence rates, fewer complications and better long-term function than the previous two techniques. Familiari et al. (40)concluded that any errant repair method for these variants may result in a significant impact on the external rotation function of the shoulder. In this study, we took a relatively simple approach to suturing the pulley ring and performed only appropriate fixation of the glenoid labrum without excessive intraoperative debridement to maintain the relative integrity of the glenohumeral joint. This study proved to be beneficial to the long-term stability of the shoulder joint through relatively long-term follow-up.

The treatment of simple second-degree SLAP injuries in patients of different ages is a controversial topic (13). Some suggest that younger patients with higher activity levels should choose glenoid labral fixation of the LHBT *in situ* instead of MDBT (41), and it has been emphasized that MDBT performs poorly in terms of long-term shoulder stability in relation to activity. However, the opposite view has also been reported, suggesting that greater motion leads to long-term instability of the LHBT and that transpositional fixation is necessary (42). Our study addressing long-term function after shoulder arthroplasty provides evidence for the treatment of MDBT in young patients with SLAP injuries. The longer-term efficacy of the modified MDBT technique in treating patients with SLAP injuries needs to be further investigated. In this study, we were not able to complete a longer-term follow-up, only for is that most of the patients had fully recovered after one year, but their adherence to follow-up decreased. However, it is certain that age is not an obstacle to the long-term efficacy of the surgery during the current follow-up period, and that the range of surgical applicability of MDBT and modified MDBT can be extended to younger patients with better long-term outcomes.

## Limitations

The limitations of this study are as follows. (i) This study was not conducted as a multi-center randomized controlled study. (ii) Sample size not large enough for subgroup analyses for each variable. (iii) Our attempts to conduct survival analyses with a return-to-work status as the endpoint event did not result in a valid analytical process. (iv) More nuanced and discriminative indicators of long-term shoulder function need to be developed.

## Conclusions

Modified MDBT may be more effective in improving long-term shoulder function and better facilitating glenohumeral joint closure. Therefore, it can be considered as a better new option for isolated type II SLAP lesions.

## Data Availability

The original contributions presented in the study are included in the article/Supplementary Material, further inquiries can be directed to the corresponding author/s.
